# Predicting Spike Occurrence and Neuronal Responsiveness from LFPs in Primary Somatosensory Cortex

**DOI:** 10.1371/journal.pone.0035850

**Published:** 2012-05-07

**Authors:** Riccardo Storchi, Antonio G. Zippo, Gian Carlo Caramenti, Maurizio Valente, Gabriele E. M. Biella

**Affiliations:** 1 Institute of Molecular Bioimaging and Physiology (IBFM), Segrate (Milan), Italy; 2 Institute of Biomedical Technologies (ITB), Segrate (Milan), Italy; Instituto de Neurociencias de Alicante UMH-CSIC, Spain

## Abstract

Local Field Potentials (LFPs) integrate multiple neuronal events like synaptic inputs and intracellular potentials. LFP spatiotemporal features are particularly relevant in view of their applications both in research (e.g. for understanding brain rhythms, inter-areal neural communication and neronal coding) and in the clinics (e.g. for improving invasive Brain-Machine Interface devices). However the relation between LFPs and spikes is complex and not fully understood. As spikes represent the fundamental currency of neuronal communication this gap in knowledge strongly limits our comprehension of neuronal phenomena underlying LFPs. We investigated the LFP-spike relation during tactile stimulation in primary somatosensory (S-I) cortex in the rat. First we quantified how reliably LFPs and spikes code for a stimulus occurrence. Then we used the information obtained from our analyses to design a predictive model for spike occurrence based on LFP inputs. The model was endowed with a flexible meta-structure whose exact form, both in parameters and structure, was estimated by using a multi-objective optimization strategy. Our method provided a set of nonlinear simple equations that maximized the match between models and true neurons in terms of spike timings and Peri Stimulus Time Histograms. We found that both LFPs and spikes can code for stimulus occurrence with millisecond precision, showing, however, high variability. Spike patterns were predicted significantly above chance for 75% of the neurons analysed. Crucially, the level of prediction accuracy depended on the reliability in coding for the stimulus occurrence. The best predictions were obtained when both spikes and LFPs were highly responsive to the stimuli. Spike reliability is known to depend on neuron intrinsic properties (i.e. on channel noise) and on spontaneous local network fluctuations. Our results suggest that the latter, measured through the LFP response variability, play a dominant role.

## Introduction

Local Field Potentials (LFPs) and spikes represent two aspects of neural signalling, tightly combined in complex causal relations [1], [2]. A better comprehension of their dynamical interactions is fundamental to provide a multi-scale picture of local sensory processing, ranging from multiple sub-threshold events to spikes. So far, since the first LFP-spike analyses, it has been possible to elucidate the spatial and temporal scales of synaptic input integration [3], [4], to improve the readout of sensory stimuli [5], [6] and to hypothesize efficient modalities of neuron-to-neuron communication between distant brain areas [7], [8]. However, the LFP-spike relation requires further clarification. In particular, only few attempts have been made to predict spike occurrence from LFP oscillations [9], [10].

In this context, our aim was to investigate the LFP-spike relation in tactile sensory system and to find simple analytical relations to predict spikes from LFPs. To carry out our investigation we performed extracellular recordings in the rat primary somatosensory cortex (S-I) in ongoing and stimulated regimes. Neurons in S-I are known to integrate a complex signal packet of temporal and modal features with millisecond precision [11]–[13].

We divided our computational analyses into two successive steps. First we quantified the accuracy of spikes and LFPs in coding for the stimulus occurrence and how they relate to each other. Then we estimated a predictive model to infer spike occurrences from simultaneous LFP recordings. Because the LFP-spike relation is highly nonlinear, the estimation of a predictive model represents a demanding computational task. To deal with this problem we developed a novel multi-objective framework based on the NSGAII algorithm [14].

We observed that the majority of spiking activity was predictable from LFPs but for a minority of cases. Crucially, we found that spike occurrence could be predicted above chance only when both LFP and spike recordings were responsive to stimuli.

## Results

### Coding for the Stimulus Occurrence: Relation Between Spike and LFP Responsiveness

In the attempt to recognize a relation between the local network level (LFPs) and the single neuron activity (spikes), we first determined stimulus responsiveness of spikes and LFPs.

We define spike responsiveness in terms of spike counts, i.e. a neuron is responsive when its spike count (number of spikes within a time window) after the stimulus onset is different from that before the stimulus. To quantify spike responsiveness by comparing the spike counts collected before and after the stimulus occurrence we used the Shannon’s Mutual Information (MI) (see Methods). MI quantifies the uncertainty reduction, about whether or not a stimulus was presented, provided by the observation of the spike response. The MI reaches its highest attainable value of 1 bit, when the spike count reduces to zero the uncertainty about stimulus occurrence.

**Figure 1:**
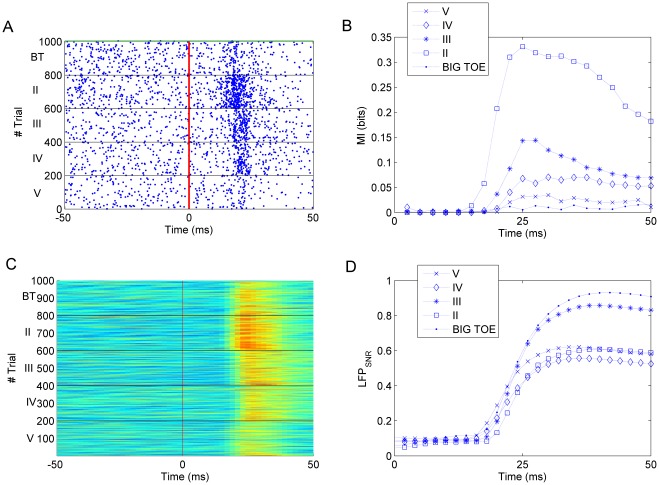
Coding for stimulus occurrence: spike and LFP responsiveness. A) Raster Plot for a single cell response to fingertip stimulation (big toe at the top and V at the bottom). Different fingertips are separated by black lines, the vertical red line indicates the time of stimulus onset. B) Mutual Information about stimulus onset for cell in (A). C) LFP response recorded simultaneously and from the same electrode of (A). D) LFP responsiveness, computed as 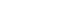.

In Fig.1A we show a neuron that responds to repetitions of five different stimuli (the five fingertips). The MI rises above 0 after 15–20 ms, peaks between 20 and 30 ms and then decreases (Fig.1B). The decrease can be related to a stimulus dependent inhibition that partially cancels out the increase in firing rate [15].

**Figure 2:**
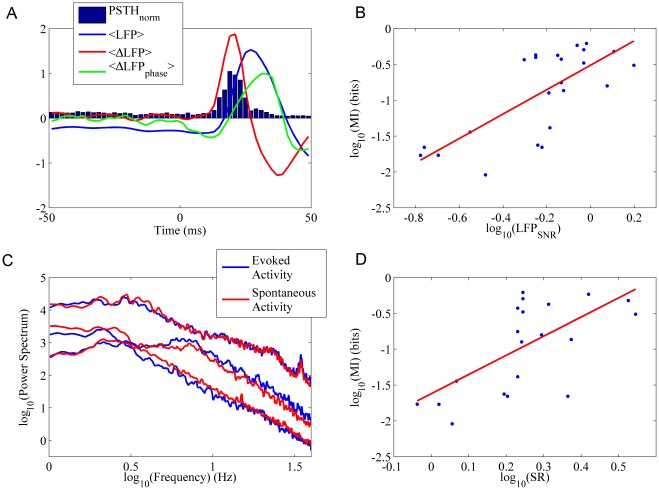
Characterization of the LFP-spike relations. A) 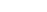 (histogram), average LFP response (blue line), average LFP derivative (red line), average phase of the LFP derivative (green line). All these responses are normalized (mean subtracted and divided by the standard deviation). B) Positive correlation between 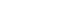 and 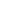. C) Power Spectrum for the spontaneous and evoked activity in three different recordings. Note that the onset of evoked activity does not impact substantially on the LFP frequency content. D) Positive correlation between 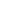 and 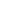.

LFPs are continuous signals and their discretization, necessary for MI estimation, poses non trivial problems [2]. We thus decided a different characterization of LFP responsiveness; namely the ratio of stimulus-dependent to stimulus-independent oscillation amplitudes (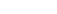, see Methods). LFP responsiveness rises after 15–20 ms and constantly increases in the considered time window of 50 ms (Fig.1C,D). Moreover not only the raw LFP but also its derivative and the phase of its derivative (see Methods) were responsive to stimulus (Fig.2A).

In the whole dataset, both LFP and spike responsiveness were highly variable, ranging between 0.17–1.58 (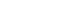) and 0.01–0.61 bits respectively. As shown in Fig.2B, the two measures were positively correlated (p = 0.003, ranksum test).

To understand if the spontaneous LFPs could provide a hint on their responsivity to stimuli, we recorded 5 minutes of spontaneous LFP activity antecedent to the stimuli. Then we computed the LFP spectrum during spontaneous and stimulated conditions and their Spectral Ratio (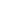, see Methods). 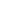 is larger (smaller) than 1 when the stimulus increases (decreases) the LFP oscillation frequency power. In two cases the stimulus increased the LFP oscillations by 5 orders of magnitude (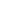 = 7.36*10^5^, 7.48*10^5^). In all the other recordings but one the oscillations were always slightly increased (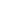 interval = 1.05 3.51, Fig.2C). Furthermore, 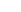 was positively correlated to spike responsiveness (p = 0.006, ranksum test, see Fig.2D).

Our results show that LFPs respond to stimuli with comparable variability to neuronal spiking, positively co-varying with them. We hypothesize that spiking variability is mainly due to a local responsive/unresponsive cortical state (see below).

### LFP-Spike Coupling Depends on Responsive/Unresponsive Cortical States

The second aim of this work is to investigate the possibility of spike prediction from simultaneous LFP recordings. The LFP-spike coupling has been shown to covary with several physiological parameters [16], [17]. Our analyses could shade light on the necessary conditions for a significant LFP-Spike coupling during tactile processing.

We reported in the previous paragraph that neuronal spikes and LFPs have correlated responsiveness. In addition to raw LFP responses we identified two other relevant signals: the LFP derivative and its phase (Fig.2A). Thus we used these three signals to predict spike occurrence.

To evaluate the goodness of prediction we selected two criteria associated with two different cost functions: a local one, based on trial-to-trial comparisons, and a global one, based on the average response. For the local cost, called Spike Match (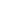), it was required that the number of incorrectly predicted 1’s and 0’s was the smallest possible. For the global cost, called PSTH Fit (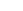), it was required that the PSTH of the model approximated that of the true neuron with the least root mean squared error (see Methods). The costs were normalized between 0 and 1 indicating respectively the best (“cheapest”) and the worst (“most expensive”) model. A cost equal to 0 implies that the model exactly matches the true neuron for that criterion. A cost equal to 1 implies that the model does not perform above chance level. Because these criteria were to some extent conflicting we obtained two set of models, one optimal for the local criterion, the other for the global one.

We found that the local criterion was harder to achieve. On average, in our dataset the optimal models for the global cost could perform 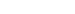 (respectively mean and standard deviation) for 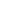 (but only 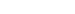 for 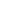), while optimal models for the global cost achieved 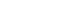 for 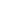 (but only 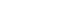 for 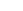). This means that models optimized for the global and the local costs could predict, respectively, 70% and 15% above chance; however, in both model sets, the best performances in one criterion came at the price of a significant worsening in the other. Overall 18 neurons (75%) were predicted above chance.

**Figure 3:**
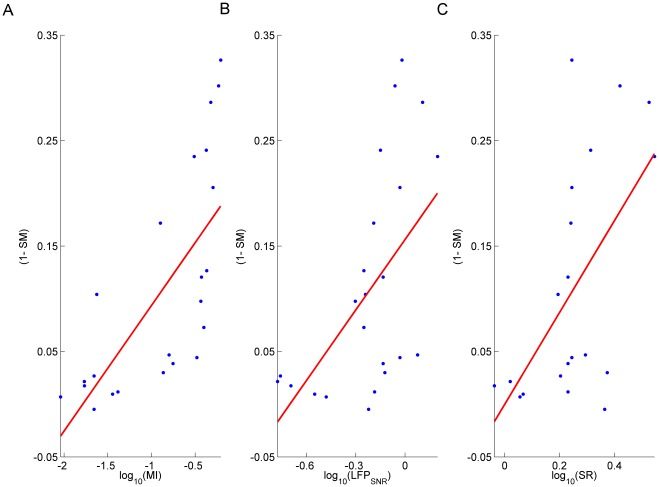
Relation between spike predictability and neuronal responsiveness. Spike predictability, quantified here with 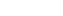, positively correlates with 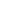 (A), 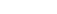 (B) and 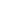 (C).

To evaluate if spike or LFP responsiveness could modulate the prediction outcomes, we first computed the correlation between the spike responsiveness and the 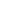 values of the optimal models for the local cost (Fig.3A). The 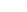 values were significantly correlated with spike responsiveness (p = 0.0006, ranksum test). Significant correlations were also revealed for 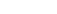 and 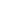 (p = 0.01, 0.04, ranksum test) as shown in Fig.3B,C. No significant correlations were found by repeating the tests with the global score 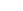 (p<0.05).

The correlation between 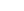/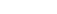/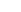 and prediction outcomes (at least for 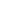) provided further support for the hypothesis that a cortical responsive/unresponsive state could significantly modulate the LFP-spike coupling.

Along our hypothesis, the cortical state was characterized by a different level of basal firing rate which positively correlated with 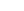 and 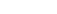 (respectively p = 0.0008 and p = 0.0084). The basal firing rate also positively (negatively) correlated with the smallest achievable 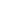 (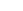) values (p = 0.0030 and p = 0.0037). The positive relationship between basal firing rate and responsiveness suggests that a cortical responsive state could emerge from a high input regime and maximize the coupling between LFPs and spikes.

### A Multi-objective Framework for the Estimation of Predictive Models

From our results on LFP-to-spike prediction we found that different evalution criteria return diverse optimal models that, interestingly, capture different aspects of the complex LFP-spike dependence. In order to reconcile these extremes we show here, by using a multi-objective strategy, that it is possible to obtain a set of models perfoming at intermediate levels in both criteria.

We used a modified version of the NSGAII algorithm [14] to optimize the prediction outcomes and find the Pareto optimal predictive models. Given the defined cost functions, these models are an estimate of the best attainable trade-off predictions [18], [19]. For each pair of Pareto optimal models if the first is “cheaper” for one criterion then, by definition, it has to be more “expensive” in at least one of the other criteria (see also Methods). In comparison with single objective strategies, multi-objective ones have the advantage to provide, together with models optimal for a specific cost, a continuum of alternative models with intermediate performances in all the diverse and potentially conflicting costs.

**Figure 4:**
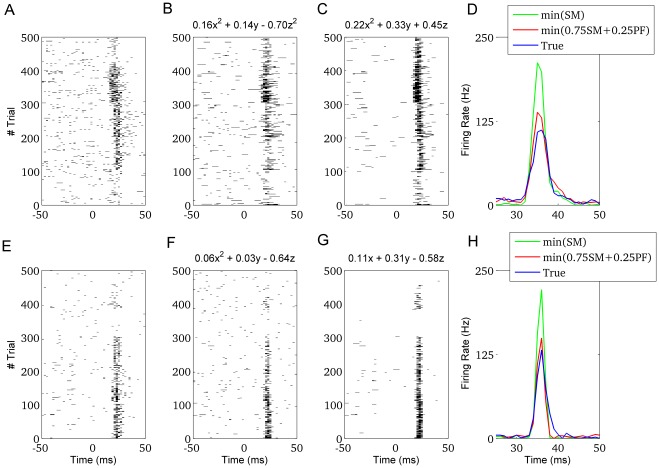
Multi-objective model optimization: extreme solutions and trade-offs. A) Binarized response for the neuron in Fig.1A. B) Predicted response for a model belonging to the optimal Pareto front. The predictive performances of this model represent a suitable trade-off between 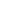 and 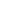. C) Predicted response for a model belonging to the optimal Pareto front. This model has the best predictive performances for 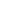 at the expense of a significant worsening in 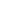. D) Average response for the true neuron (blue) and the model in (B) and (C) (respectively red and green lines). E-H) Same as (A-D) for a different cell. For the model in (F) 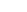 and 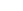 are respectively 0.70 and 0.41. For the model in (G) 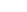 = 0.64 and 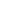 = 0.61. The smallest 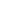 achievable was 0.36 (but for 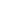 = 0.74).

As representative case we report (Fig.4A) the binarized response of the neuron in Fig.1A. We show, in Fig.4B,C, the predictions from two Pareto optimal models, the first minimising the composite cost 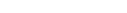, the second minimising the local cost 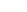. Although the latter scored the largest number of correct 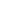
’s and 0’s predictions (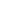 = 0.72), it largely failed to capture the PSTH of the true neuron (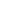 = 0.61, Fig.4D). The best model for the composite index represented a trade-off between the local and the global criteria (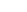 = 0.78, 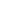 = 0.34). The optimal model for the PSTH prediction (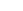 = 0.20, not shown) was achieved at the price of a substantial worsening in 1’s and 0’s prediction (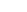 = 0.84). A different neuron, whose models have similar prediction outcomes, is reported in Fig.4E–H (model performaces are reported in captions).

The mathematical laws associated with the different models are reported at the top of each panel (Fig.4B,C,F,G), where 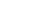 represent respectively the LFP signal, its derivative and the phase of its derivative. To the best of our knowledge this is the first time the LFP-to-spike trasformation is formalized into simple deterministic laws derived from experimantal data.

## Discussion

LFPs are signals integrating a number of variable electrical events, the population synaptic activity in the first place [1], [20], [21], while spikes account for selected and stereotyped occurrences. This mapping of “many to ones” is a supposedly finely regulated registry granting for tuned signal encoding and decoding. How this LFP-to-spike transition takes place is, however, largely unknown [16] and deserves further investigation.

In this perspective, we analysed the cortical neuronal responses to non-noxious light tactile stimuli, trying to inspect simple rules for the LFP-to-spike transition during cortical input processing. We thus chose a mechano-vibrating stimulus (see Methods), likely to recruit deep skin receptors (e.g. Pacinian bodies [22], [23]) with high temporal and poor spatial resolution and cortical neurons with large receptive fields in laminae II to IV.

We first showed that spikes and LFPs code for the stimulus occurrence, although both with variable levels of reliability. Then we found that, by estimating a simple analytical model, spikes can be predicted significantly in 75% neurons. Only in the presence of jointly responsive LFPs and spikes could we estimate sufficiently accurate analytical rules for their dependence.

The LFP-spike relation typically reflects local functional connectivity, as shown e.g. for the visual stimuli in different cortical areas [3], [46]. Our findings corroborates the increasingly accumulating evidence that the modulation of local functional connectivity, reflected in a variable LFP-spike relation, critically affects the spike response. A network whose spike responses are modulated by functional connectivity has at least two great advantages: it can create dynamical associations (e.g. for multisensory integration) and it is more robust to network failures.

At present experimental evidences reported a composite picture of the LFP-spike relations associated with the coding of sensory information. Single neurons have been found capable of locking their spikes both to local and to distant LFPs [8]. Low frequency LFPs and spikes have been shown to convey non redundant sensory information [5] and spike probability seems to be influenced by the local LFP phase [6], [7] and by LFP amplitudes. The strenght of LFP-spike relation also reflects the level of surplus (non-poissonian) spike synchrony [16], [17]. Coherently with this complex scenario, we observed, in S-I cortex, that spike occurrence is influenced at least by three different LFP features: the LFP amplitude, the LFP derivative and its phase.

Several works have reported a high variability in the level of regional coupling between LFPs and spikes [16], [24]. We also found that the strenght of their relation was largely variable. LFPs also correlate with the neuronal membrane potential [1]. Simultaneous intra- and extracellular recordings reported that the LFP couplings with spikes and membrane potentials positively covary [25].

We observed that both spike responsiveness and spike predictability were significantly correlated with the basal firing rates. To the extent the firing rates we recorded were proportional to the number of synaptic inputs (this relation was not necessary monotonic [26], [27]), elevated basal rates could be associated with a high input regime. This cortical condition was proposed to be optimal for stable information propagation within the cortex [28]. Our results may suggest that a cortical responsive state could emerge from a high input regime and maximize the coupling between LFPs and spikes.

LFP responses are likely to mainly represent recurrent cortical activity (thalamic inputs represent only a small fraction of synapses in layer IV cortex [29]). Cortical amplification through recurrency is a well known mechanism, at least in visual cortex [30], [31], and could play a key role in LFP-spike coupling.

Few attempts have been made so far to predict spikes from LFPs [9], [10]. For the first time we propose a computational framework that returns a relatively simple, although flexible and nonlinear, analytical model. For the estimation of the structure and the parameters of our model we relied on a slightly modified version of the NSGAII algorithm [14]. NSGAII has been successfully used for a variety of purposes, ranging from optimal parameter estimation of Proportional Integral Derivative (PID) controllers, to laser tuning in quantum optimization, robot design and trajectory planning. [32]–[37]. At the best of our knowledge, this is the first time that this is used for predictive modelling of neuronal responses.

The estimation of a predictive model for spikes implies the implicit assumption of a spike metric, i.e. a distance measure to compare true and predicted spike patterns. The calculation of a spike metric has been proved a computational demanding task and relies on *a priori* hypotheses about the most salient features of a spike pattern [38]. Most predictive models avoided this complicacy assuming that a single cost function based on the average response could return the best model (e.g. [39], [40]). We show that this is not necessary the case and the joint evaluation of the average response and of a local measure, based on trial-to-trial comparisons, could return a more complete set of models that capture diverse aspects of the true responses.

The analytical relations we estimated with our predictive framework do not directly imply a causal relation between LFP and spikes. The direction of the LFP-spike interaction was investigated for V1 cortex in a recent work [2] and the authors unveiled a complex scenario of both symmetric and asymmetric dependences.

We asked how and to what extent the occurrence of a stimulus modified the LFP activity. We found that the stimulation had a small but significant effect and LFP oscillations were slightly enhanced. The size of this effect was positively correlated with LFP and spike responsiveness and spike predictability.

In conclusion, our results suggest that the LFP-spike relation could shed light on the functional states (e.g. responsiveness/unresponsiveness) of cortical circuits. Indeed, we showed that LFPs are good predictors for spikes whenever neurons are responsive to stimuli. Our results on prediction, besides the theoretical interest, could also potentially improve the current strategies for programming efficient neuroprosthetics [41].

## Methods

### Ethical Statement

To study how sensory stimuli are represented by neuronal activity there is no alternative to the use of animals and the use of an in vivo approach. The animals were maintained with regulated 16 hrs light- 8 hrs dark cycles, food and water ad libitum. All the animals have been treated according to the Italian and European Laws on animal treatment in Scientific Research (Italian Bioethical Committee, Law Decree on the Treatment of Animals in Research, 27 Jan 1992, No. 116).

The National Research Council, where the experiments have been performed, adheres to the International Committee on Laboratory Animal Science (ICLAS) on behalf of the United Nations Educational, Scientific and Cultural Organizations (UNESCO), the Council for International Organizations of Medical Sciences (CIOMS) and the International Union of Biological Sciences (IUBS). As such, no protocol-specific approval was required.

### Electrophysiological Recordings

Fifteen male rats (Sprague-Dawley, Charles River, Calco, LC, Italy) weighing 300–400 g were used. The neuronal electrophysiological recordings were taken from the side contralateral to the stimulated paw. A 3 mm^2^ hole was drilled on the skull to gain access to the Somatosensory Primary (S1) cortex. The neuronal recordings were obtained by a vertical multitrode with 8 gold contacts, 7 located on a linear array (125 µm contact spacing) and one on the tip (tip-first array distance was 370 µm). The average impedence was 1.8 MΩ (Thomas RECORDING GmbH, Giessen, Germany). Brush light tactile stimuli were delivered onto the plantar aspect of the left hindlimb to assess the somatotopic correspondance with the sciatic innervation field.

The rats underwent preliminary barbiturate anesthesia for the surgical experimental preparation. The jugular vein and the trachea were cannulated to gain, respectively, a drug delivery pathway and the respiratory connection to the anaesthesia-ventilation device. After the preparatory stage, the rats were mounted on a stereotaxic apparatus (Narishige, Tokyo, Japan) and a bone tile was excised on the hindlimb cortical representation area. An electronically regulated thermal bed maintained the rat temperature at 37.5 Celsius degrees. After drilling a bone tile on the skull giving access to the posterior paw somatosensory projection area [42], the dura mater was delicately removed and the cortical electrode inserted.

Before its placement, the rats were paralyzed by intravenous gallamine thriethiodide (20 mg/kg/h) injection and connected to an automatic respiratory device delivering (1stroke/s) Isoflurane 2.5% 0.4 to 0.8 l/min and Oxygen 0.15–0.2 l/min gaseous mixture. Curarization was maintained stable throughout the whole experiment by Gallamine refracted injections. During the experiment the anaesthesia level was continuously monitored by 4 EEG channels. The EEG electrodes were placed controlaterally to the cortical electrode, along a fronto-occipital sequence.

For signal amplification and data recordings, we used a 32 channel Cheetah Data Acquisition Hardware (Neuralynx, MT, USA) at 32 kHz sampling frequency. Electrophysiological signals were acquired between 1 Hz and 6 kHz. The data were stored for offline analyses. A histological confirmation of the placement of the electrodes was then obtained on brain coronal sections stained with cresyl violet.

### Stimulation Protocol and Preliminary Analyses

Brush light tactile stimuli were delivered onto the plantar aspect of the left hind limb to assess the correct somatotopy by cortical responsiveness to the sciatic innervation field. The repetitive and preserved responses to stimuli were the anatomo-functional acceptance criteria for data acquisition. Controlled stimulation was delivered through a blunted cactus tip. The tip was mounted on the dust cap of a speaker and driven through a microcontroller board (Arduino [43]). At the beginning of each stimulation epoch the tip was lightly placed over the skin. Fast 5 ms pressure pulses were applied following a semi-random sequence. Pulses occurred in couplets. The delay between every first pulse of each couplet was equal to 500 ms. Every second pulse followed the first by a random delay extracted uniformly in the range between 50 and 250 ms.

The extracellular recordings were numerically filtered in the band 1–100 Hz and 300–6000 Hz to obtain respectively Local Field Potentials (LFPs) and spikes. LFPs were then downsampled to 0.5 KHz. We used the same techniques for filtering as described in [6]. After filtering and downsampling the spike contamination of LFP signal was null, so no further spike removal techniques [44] were needed. The spikes were extracted and sorted by using internally developed software. Time bins, to compute the match between observed and predicted neuronal responses, were set at 2 ms. The LFP signal was convolved with the spike-triggered average. From the latter we extracted two additional signals: the LFP derivative and the phase of the LFP derivative. The LFP phase was computed by using the Hilbert transform (implemented in Matlab by the function *hilbert.m*).

### Evaluation of Spike Responses

The stimulus responsiveness for single units was computed by using the Shannon’s Mutual Information (MI). The conditional response probability 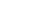 was estimated from the neuronal response, 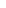 representing the stimulus category, 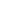 the number of spikes emitted within a fixed time window (i.e. the spike count). The stimulus category was either 1 (stimulus) or 0 (no stimulus). Because the two categories were equiprobable the largest value for MI was 1 bit. The distribution 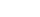 was obtained from the spike counts in time window starting, in each trial, at the time of stimulus deliverance. The distribution 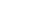 was obtained from the spike counts in a time window of equal duration and ending at the time of the stimulus deliverance. To characterize the neuronal responsiveness in each location we used the following definition of MI:
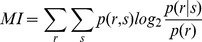
(1)In order to find the optimal time window we repeated the measure with time windows of increasing size (from 5 to 50 ms at steps of 5 ms). The search for the optimal time window was motivated by the fact that neuronal responses typically exhibited a complex two-phases behaviour: the firing rate initially increased over the basal level and, after 20–40 ms, decreased under the basal level. The timing of phase switch was unit-dependent. The stimulation was applied in 5 different locations on the hindpaw and for each location 200 stimulus repetitions were delivered. After evalution across the different stimulus locations and time windows we took the largest MI value obtained.

To obtain unbiased estimates of the Mutual Information we used a procedure described in [45]. Following the authors prescriptions the plug in estimate of 1 was corrected by using two additional terms: the shuffled entropy 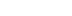 and the independent entropy 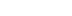. The resulting corrected estimator has been shown to converge to the correct value much faster than the associated plug in estimator [45].

As the corrected estimator is obtained by using a shuffling procedure its value is slightly different every time the estimation is repeated. To counterbalance for those random fluctuations we took the average of 100 repetitions, its standard deviation representing the level of intrinsic noise in the measure. Accordingly the MI was considered reliable only if its noise was less than 5% of the smallest marginal entropy. For all the reported MI estimates the noise level was under this threshold.

### Evaluation of LFP Responses

The stimulus responsiveness for LFPs was evaluated by using a measure of the signal-to-noise ratio. To this aim we defined a response matrix 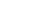. 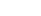 is a single trial response sampled at different times from the stimulus onset. 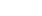 collects the responses to all trials sampled at a specific delay from the stimulus onset. We called 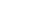 the mean of 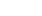 and 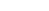 the mean of 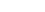
s over all possible delays. We also defined 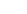 the number of response samples in a trial and 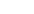 the overall number of trials. Then the LFP responsiveness, 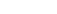, was measured as
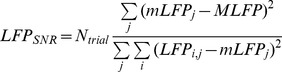
(2)where 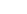 cancels out in the division. The numerator quantifies the mean stimulus-evoked response after removal of the intrinsic, stimulus-independent fluctuations while the denominator quantifies the size of such fluctuations. When trial-to-trial fluctuations are much larger than the evoked response, then the LFP responsiveness tends to 0. Conversely, when the evoked response dominates, 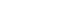 tends to a large value.

A different measure of LFP responsiveness was based on the spectral content of the spontaneous and the stimulated LFP. In order to quantify the frequency-wise distance, we used an index called Spectral Ratio (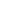)
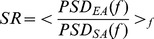
(3)where 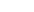 represents the Power Spectral Density. The spectral frequency 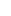 was evaluated in the interval 1–39.9 Hz with steps of 0.3 Hz.

### Model Design

We defined a class of models that took as input different LFP features returning a binary output, 1 for spike 0 otherwise. From the LFP (convolved with the spike-triggered average) we extracted two additional features: the LFP derivative and the phase of the LFP derivative (see Fig.2A). Then the LFP, the LFP derivative and its phase were normalized (subtracted by the mean and divided by the standard deviation) and used as model inputs.

Let us call 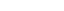 these inputs. We define a weight sequence 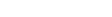 (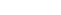 for any 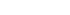 and 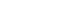) and a set of unary operators 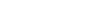, where 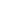 belongs to an operator set 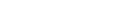. We define the model structure 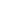 as a 3-ary operator
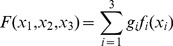
(4)A combination of the 3 unary operators is chosen among the 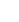 possible ones. The 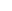 operator defines, through the operators 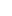 and the weights 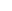, respectively the structure and the parameters of the predictive model. We convert a neuronal signal into a binary one, where 1 occurs at the time of spike emission and 0 otherwise. Let 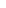 and 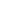, respectively, the binary signals associated with a real neuron and its predicted activity. To convert 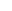 into a binary signal we applied a threshold 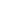 so that the full LFP-spike transformation can be expressed as
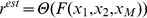
(5)and 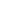 represents the Heavyside function (equal to 1 when the argument is positive, zero otherwise) with threshold 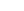. As we chose 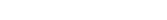, the model structure was evaluated within a set of cardinality 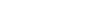. To clarify with an example, if we select an operator combination 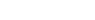 and a weight sequence 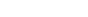, then we define the model as 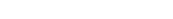 and the LFP-to-spike transformation as 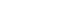.

The 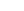 value was set so that the number of spikes emitted by the model response 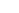 was equal to that detected for the true response 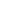. We called the latter 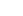. Given the three inputs 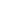, 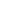, 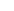 the full search space for the model structure is represented by the following expression
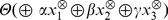
(6)where we set 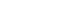 and 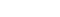.

### Model Optimization Criteria

To evaluate the goodness of a model we need to quantify the error in prediction represented by the distance between predicted and true spike trains. The definition of an appropriate distance measure for spike patterns, i.e. of a spike metric, needs to incorporates different pattern features like the timing and the number of spike occurrences [38]. Given the multidimensional aspects of the optimization task we decided to take into consideration two main criteria to evaluate model predictions. The first was local and based on trial-to-trial measurements, the second was global and based on the distance between the true and the average response. The local objective was called Spike Match (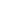) and computed with the following expression.
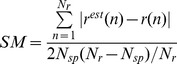
(7)where 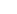 represents the length of the response vectors. The denominator was purposedly added in order to obtain 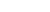 for predictions at chance level. Its derivation can be found in Supplementary Information S1. The response distance 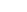 represents the local objective to minimize.

The global objective was called 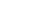 Fit (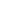). It was computed as follows
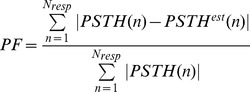
(8)We used this criterion to test whether a neuron response was predicted above chance. We counted as above chance prediction any neurons whose average 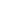 value subtracted by the standard deviation (in the first Pareto Front) was less than unity. The best predictions would be obtained by minimising both 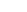 and 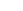 (ideally 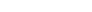, although, for our experimental data, this never happens). Note that there is no guarantee that models with 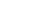 will also have 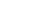. However if 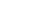 (i.e. the model can predict all spike/non spike occurrences), then 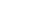. If 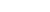 there is no rule on 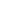 values.

According to *Ockham’s Razor principle* we used a third objective function to incorporate a measure of complexity within the optimization process. We elected as simplest possible a model with 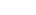, the others being all zeros. Operatively, we tried to minimize a third objective, the Complexity Order (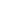), expressed as
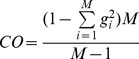
(9)In the most complex case (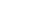) 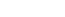 for each weight.

### Optimization Algorithm

In most practical situations multiple objective frameworks may be preferable [18], [19] over single objective ones. To find the best solutions, we used the framework of Pareto non-dominated sorting. Given two candidate solutions 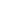, 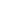 and their objective functions 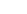 and 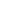, we say that 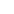 dominates 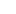 when 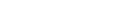 for any 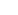 objective and at least one of those comparisons returns a strict inequality. If both 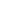 does not dominate 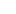 and vice versa, we say that 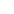 and 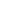 belong to the same front of non-dominated solutions.

Among the wide family of algorithms based on Pareto front evaluation, we chose the well known Non-Dominated Sorting Genetic Algorithm II (NSGAII) [14]. The initial population was generated by randomly sampling from structure and parameter space. The population size was chosen equal to 320 so that each structure was represented, on average, by 5 individuals. Two parents were mated only when they exhibited the same structure. This condition (restricted mating, not present in the original NSGAII) allows for the generation of offspring only through parents sharing the same structure.

When mating occurs, crossover is implemented on parameters and each weight 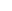 is extracted with equal probability from one of the parents. Weights from the selected parent are copied into the child genotype. Then, they are modified in order to constrain the weight sum to equal unity. Mutations occur with probability 0.1 both on the structure and on the parameters, respectively by switching an operator or by adding a Gaussian 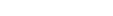 variation to the selected weight.

The algorithm exhibited no substantial improvement after 10–20 iterations. Accordingly, we fixed 50 iterations as stop criterion. At the end of a run, the algorithm always selected few structures and, for each, they converged onto 1–2 regions of parameter combinations.

Each dataset was divided into two subsets of equal size, the first was used for the training phase, the second for the cross-validation. The Pareto optimal solutions, extracted from the training set, were then evaluated on the cross-validation set. All non-dominated solutions in the latter set were finally selected.

## Supporting Information

Supplementary Information S1 SM Normalization
(PDF)

## References

[1] Pesaran B (2009). Uncovering the Mysterious Origins of Local Field Potentials.. Neuron.

[2] Besserve M, Schlkopf B, Logothetis NK, Panzeri S (2010). Causal relationships between frequency bands of extracellular signals in visual cortex revealed by an information theoretic analysis.. J Comput Neurosci.

[3] Nauhaus I, Busse L, Carandini M, Ringach DL (2009). Stimulus Contrast Modulates Functional Connectivity in Visual Cortex.. Nat Neurosci.

[4] Belitski A, Panzeri S, Magri C, Logothetis NK, Kayser C (2010). Sensory information in local field potentials and spikes from visual and auditory cortices: time scales and frequency bands.. Journal of Computational Neuroscience.

[5] Belitski A, Gretton A, Magri C, Murayama Y, Montemurro M (2008). Low-Frequency Local Field Potentials and Spikes in Primary Visual Cortex Convey Independent Visual Information.. J Neurosci.

[6] Montemurro MA, Rasch MJ, Murayama Y, Logothetis NK, Panzeri S (2008). Phase-of-Firing Coding of Natural Visual Stimuli in Primary Visual Cortex.. Current Biology.

[7] Fries P (2005). A mechanism for cognitive dynamics: neuronal communication through neuronal coherence.. Trends Cogn Sci.

[8] Canolty RT, Ganguly K, Kennerley SW, Cadieu CF, Koepsell K (2010). Oscillatory phase coupling coordinates anatomically dispersed functional cell assemblies.. Proc Natl Acad Sci U S A.

[9] Rasch MJ, Gretton A, Murayama Y, Maass W, Logothetis NK (2008). Inferring spike trains from local field potentials.. J Neurophys.

[10] Galindo-Leon EE, Liu RC (2010). Predicting stimulus-locked single unit spiking from cortical local field potentials.. J Comput Neurosci.

[11] Foffani G, Chapin JK, Moxon KA (2008). Computational Role of Large Receptive Fields in the Primary Somatosensory Cortex.. J Neurophysiol.

[12] Maravall M, Petersen RS, Fairhall AL, Arabzadeh E, Diamond ME (2007). Shifts in Coding Properties and Maintenance of Information Transmission during Adaptation in Barrel Cortex.. PLoS Biol.

[13] Petersen RS, Brambilla M, Bale MR, Alenda A, Panzeri S (2008). Diverse and Temporally Precise Kinetic Feature Selectivity in the VPm Thalamic Nucleus.. Neuron.

[14] Deb K, Agrawal A, Pratab A, Meyarivan T (2000). A fast elitist nondominated sorting genetic algorithm for multi-objective optimization: NSGA-II.. IEEE Transaction on Evolutionary Computation.

[15] Gabernet L, Jadhav SP, Feldman DE, Carandini M, Scanziani M (2005). Somatosensory integration controlled by dynamic thalamocortical feed-forward inhibition.. Neuron.

[16] Nir Y, Fisch L, Mukamel R, Gelbard-Sagiv H, Arieli A (2007). Coupling between Neuronal Firing Rate, Gamma LFP, and BOLD fMRI Is Related to Interneuronal Correlations.. Current Biology.

[17] Denker M, Roux S, Linden H, Diesmann M, Riehle A (2011). The Local Field Potential Reflects Surplus Spike Synchrony.. Cereb Cortex.

[18] Jin Y (2006). Multi-objective machine learning..

[19] Knowles J, Corne D, Deb K (2008). Multiobjective Problem Solving from Nature..

[20] Mitzdorf U (1985). Current Source Density Method and Application in Cat Cerebral Cortex: Investigation of Evoked Potentials and EEG Phenomena.. Phys Rev.

[21] Anastassiou CA, Perin R, Markram H, Koch C (2011). Ephaptic coupling of cortical neurons.. Nat Neurosci.

[22] Johansson RS, Birznieks I (2004). First spikes in ensembles of human tactile afferents code complex spatial fingertip events.. Nat Neurosci.

[23] Delmas P, Hao J, Rodat-Despoix L (2011). Molecular Mechanisms of Mechanotrasduction in Mammalian Sensory Neurons.. Nat Rev Neurosci.

[24] Logothetis NK, Pauls J, Augath M, Trinath T, Oeltermann A (2001). Neurophysiological investigation of the basis of the fMRI signal.. Nature.

[25] Okun M, Naim A, Lampl I (2010). The Subthreshold Relation between Cortical Local Field Potential and Neuronal Firing Unveiled by Intracellular Recordings in Awake Rats.. J Neurosci.

[26] Kuhn A, Aertsen A, Rotter S (2004). Neuronal Integration of Synaptic Input in the Fluctuation-Driven Regime.. J Neurosci.

[27] Luccioli S, Kreuz T, Torcini A (2006). Dynamical response of the Hodgkin-Huxley model in the high-input regime.. Phys Rev E.

[28] Shadlen MN, Newsome WT (1998). The variable discharge of cortical neurons: implications for connectivity, computation, and information coding.. J Neurosci.

[29] Lefort S, Tomm C, Sarria JC, Petersen CC (2009). The excitatory neuronal network of the C2 barrel column in mouse primary somatosensory cortex.. Neuron.

[30] Douglas RJ, Koch CK, Mahowald M, Martin KA, Suarez HH (1995). Recurrent Excitation in Neocortical Circuits.. Science.

[31] Chance FS, Nelson SB, Abbott LF (1999). Complex cells as cortically amplified simple cells.. Nat Neurosci.

[32] Pedersen GK, Yang Z (2006). Multi-objective PID-controller tuning for a magnetic levitation system using NSGA-II..

[33] Bonacina L, Extermann J, Rondi A, Boutou V, Wolf JP (2007). Multi-Objective Genetic Approach for Optimal Control of Photo-induced Processes.. Phys Rev A.

[34] Saravanan R, Ramabalan S, Ebenezer NG, Natarajan R (2008). Evolutionary bi-criteria optimum design of robots based on task specifications.. The International Journal of Advanced Manufacturing Technology.

[35] Saravanan R, Ramabalan S (2008). Evolutionary Minimum Cost Trajectory Planning for Industrial Robots.. Journal of Intelligent and Robotic Systems.

[36] Gollub C, de Vivie-Riedle R (2009). Multi-objective genetic algorithm optimization of 2D- and 3D-Pareto fronts for vibrational quantum processes.. New J Phys.

[37] Kumar CA, Nai NK (2010). NSGA-II Based Multiobjective PID Controller Tuning for Speed control of DC Motor Drives.. Int J Soft Computing.

[38] Victor JD, Purpura K (1997). Metric-space analysis of spike trains: theory, algorithms, and application.. Network.

[39] Chichilnisky EJ (2001). A simple white noise analysis of neuronal light responses.. Network: Comput Neural Syst.

[40] Geffen MN, de Vries SE, Meister M (2007). Retinal Ganglion Cells Can Rapidly Change Polarity from Off to On.. PLoS Biol.

[41] Lebedev MA, Nicolelis MA (2006). Brain machine interfaces: Past, present and future.. Trends Neurosci.

[42] Paxinos G, Watson C (2006). The Rat Brain in Stereotaxic Coordinates..

[43] Arduino website. Duemilanove, Italy.. http://arduino.cc.

[44] Zanos TP, Mineault PJ, Pack CC (2010). Removal of spurious correlations between spikes and local field potentials.. J Neurophysiol.

[45] Panzeri S, Senatore R, Montemurro MA, Petersen RS (2007). Correcting for the sampling bias problem in spike train information measures.. J Neurophysiol.

[46] Hwang EJ, Andersen RA (2011). Effects of visual stimulation on LFPs, spikes, and LFP-spike relations in PRR.. J Neurophysiol.

