# Supplementary material for: Predicting Spike Occurrence and Neuronal Responsiveness from LFPs in Primary Somatosensory Cortex
Source: PLoS One. 2012 May 7;7(5):e35850. doi: 10.1371/journal.pone.0035850 (PMC3346760; doi:10.1371/journal.pone.0035850)
Supplement: Supplementary Information S1 — SM Normalization (PDF) [file pone.0035850.s001.pdf]

S1 SM Normalization

We call $\mathbf{r}$ a binary response vector from a recorded cell and $r(n)$ the response at the nth bin. Then we call $r_{est}(n)$ the estimated response in the same bin. For the training dataset we set a threshold to our model so that

$$\sum_{n=1}^{N_r} r(n) = \sum_{n=1}^{N_r} r_{est}(n) = N_{sp} \tag{1}$$

Where N_r is the number of response bins and N_{sp} is the total number of spikes emitted by the cell. We call $p_M = \frac{N_{sp}}{N_r}$ the model probability of firing a spikes. The cell firing probability depends on the time bin considered and we call $p_C(n)$ the firing probability for the nth bin and $\hat{p_C}$ the average firing probability. Note that $\hat{p_C} = p_M$.

In order to evaluate the fit of the model we propose the following index

$$SM = \frac{\sum_{n=1}^{Nr} |r(n) - r_{est}(n)|}{C} \tag{2}$$

Where C is the normalization constant expressed as

$$C = \frac{2N_{sp}(N_R - N_{sp})}{N_R} \tag{3}$$

So that the expected value for SM is 1 when the model is totally uncorrelated to cell activity. We derived C as follows:

- Let $\hat{p_{C0}}$ the average joint probability of one spike from the cell and no spike from the model
- Let $\hat{p_{0M}}$ the average joint probability of no spike from the cell and one spike from the model
- The expected value for $\sum_{n=1}^{Nr} |r(n) - r_{est}(n)|$ when model predicts by chance is equal to $N_r(\hat{p_{C0}} + \hat{p_{0M}})$

Then we compute $\hat{p_{C0}}$ and $\hat{p_{0M}}$:

$$\hat{p_{C0}} = \sum_{n=1}^{N_r} p_C(n)(1 - p_M) = \hat{p_C}(1 - p_M) = p_M(1 - p_M) = \frac{N_{sp}(N_R - N_{sp})}{N_R^2} \tag{4}$$

$$\hat{p_{0M}} = \sum_{n=1}^{N_r} (1 - p_C(n))p_M = (1 - \hat{p_C})p_M = p_M(1 - p_M) = \frac{N_{sp}(N_R - N_{sp})}{N_R^2} \tag{5}$$

Then we can obtain C as

$$C = N_r(\hat{p_{C0}} + \hat{p_{0M}}) = N_R 2\hat{p_{C0}} = \frac{2N_{sp}(N_R - N_{sp})}{N_R} \tag{6}$$
